# Fuzzy analytic hierarchy process‐based risk priority number for risk assessments of commissioning process of a ring gantry LINAC

**DOI:** 10.1002/acm2.13760

**Published:** 2022-08-23

**Authors:** Jina Chang, Siyoung Jang, Ron Lalonde, Saiful M. Huq

**Affiliations:** ^1^ Department of Radiation Oncology UPMC Hillman Cancer Center and University of Pittsburgh School of Medicine Pittsburgh Pennsylvania USA; ^2^ Department of Radiation Oncology Boston Medical Center and Boston University School of Medicine Boston Massachusetts USA

**Keywords:** analytic hierarchy process, commissioning process, fuzzy risk priority number, ring gantry LINAC

## Abstract

**Purpose:**

We propose a fuzzy analytic hierarchy process (AHP)‐based risk priority number (RPN) method in failure modes and effects analysis (FMEA) to overcome the shortcomings of traditional RPN‐based FMEA. Our research group has previously published the FMEA to mitigate the failure modes (FMs) for the commissioning process of a ring gantry LINAC. However, inter‐relationships among FMs were observed in high ranked FMs due to a heavy reliance on imaging system.

**Methods:**

Fuzzy AHP was applied to determine relative weights of risk impacts based on inter‐relationships among FMs. Since the time sequence dependency is a major factor for risk factors, a hierarchical structure of AHP was used to reflect the directional impacts such as causal influence and feedback loop. Two fuzzy weighted RPNs, called (*RPN_W_* and *FRPN_W_*, were calculated depending on the input values of severity (S), occurrence (O), and probability of not being detected (D) from the evaluators. The *RPN_W_* used numerical values, whereas the fuzzy values were used for *FRPN_W_*. Both RPNs were calculated by multiplying the weighted O, S, and D using the fuzzy AHP method.

**Results:**

The differences between the two fuzzy RPN rankings are due to inherent fuzzy uncertainty and deviations in O, S, and D values submitted by the evaluators. Considering all results of traditional and fuzzy‐based FMEA, the two most highly ranked FMs were identified: errors in determining the non‐isocentric SSD and SSD from MV images because of the unique features of the ring gantry LINAC.

**Conclusion:**

This study has demonstrated the feasibility of the use of a fuzzy AHP‐based RPN to perform comprehensive analysis and prioritization of FMs. The risk analysis using fuzzy AHP can be improved and/or refined based on the department's specific workflow and clinical preferences taking various priority weighting approaches into account.

## 1 INTRODUCTION

Failure modes and effects analysis (FMEA) is a step‐by‐step approach for identifying all possible failure modes (FMs) in order to mitigate their effects. FMEA can be done by identifying and evaluating the potential failures in a process and its effects, determining and prioritizing the risks associated with different FMs, and identifying actions that could minimize the probability of potential failures. FMEA is actively used in medical physics area to assess the risks for commissioning the LINAC,^1^ Gamma Knife,^2^ tomotherapy,^3^ brachytherapy,^4^, ^5^ implementing SRS programs,^6^ and verifying new treatment techniques^7^, ^8^ using guidance from the AAPM TG‐100 report.^9^ The risk priority number (RPN) is used as the prioritization of possible FMs in FMEA analysis. The three factors used in the calculation of RPN are severity (S), occurrence (O), and probability of not being detected (D). It is calculated by multiplying the value of S, O, and D, and can range from 1 to 1000. However, there are several shortcomings^10^, ^11^, ^12^, ^13^ of traditional RPN‐based FMEA, some of which are as follows: (1) The relative importance of the risk factors was not considered as they are accepted equally important. It can cause an identical RPN value for different combinations of O, S, and D. FMs with an identical RPN may correspond to different risk factors. (2) The values of O, S, and D show considerable discrepancies among evaluators. (3) RPNs are heavily distributed at the bottom of the scale and there are many duplicate scores for RPN in most clinical cases. The 90% of possible S, O, and D combinations result in RPN scores less than 405 because no priority is given to risk factors.^9^

A modified approach to FMEA has been introduced to overcome some shortcomings of the traditional RPN‐based failure prioritization. Healthcare failure mode and effect analysis (HFMEA) is one of the methods to use specifically in the healthcare setting.^14^ It incorporates a decision tree algorithm and hazard scoring matrix which consists of severity and frequency to evaluate FMs. The hazard score is determined by assigning severity and probability ratings of FMs based on a four‐level severity of occurrence guideline for failures (minor, moderate, major, and catastrophic) and looking up the result on the HFMEA hazard matrix. The hazard score replaces the RPN used in traditional FMEA. HFMEA has been applied for the analysis of a radiotherapy clinical information system^15^ and time‐ and cost‐saving modifications of HFMEA in radiotherapy.^16^ A comparison study of FMEA and HFMEA was also performed for quality management for surface image guided LINAC‐based radiosurgery.^17^ However, HFMEA experts considered the four‐level scoring system and descriptions recommended by the technique did not provide for a sufficient specification. Therefore, a scale description of the hazard scoring matrix always needs to be adjusted and the scale specification needs to be standardized based on selected process and the aim of the analysis. In the risk evaluation of both FMEA and HFMEA, evaluations by evaluators play a very important role. It has been argued that the risk factors are not easily evaluated and are subjective depending on the experts’ previous knowledge and experience. It inevitably involves various types of uncertainties and inconsistencies in a risk assessment.

Fuzzy theory^18^, ^19^ is another approach used to resolve defects of the traditional FMEA and HFMEA methods. Fuzzy logic is used to model complex systems with insufficient knowledge or inaccurate data. Compared with traditional RPN, the fuzzy method has the following advantages: (1) risk factors are evaluated using linguistic variables, which makes it easier for evaluating FMs; (2) relative importance of the risk factors can be considered; (3) uncertainty can be modeled by considering the probability of risk factors as a fuzzy set, instead of crisp numbers. In fuzzy theory, crisp number is used in contrast with fuzzy number. A fuzzy number has a range of values while a crisp number has a precise value. Fuzzy rule‐based FMEA method has been used to analyze accidental events during high dose rate brachytherapy treatments.^20^ It is designed to avoid highly subjective and inconsistent expert judgements and assign relative importance of the risk factors based on a fuzzy rule assessment model for calculating fuzzy RPN values. However, the construction of fuzzy if–then rules is not an easy task and requires a vast amount of expert knowledge and time.

Recently, this fuzzy theory became a powerful tool when it was combined with multiple criteria decision‐making (MCDM) methods for a risk assessment. Analytic hierarchy process (AHP)^21^, ^22^, ^23^ is one of the well‐known MCDM methods for prioritizing in the form of a hierarchy of independent elements. In traditional FMEA, failure modes are assumed to be independent of one another while there are inter‐relationships among FMs in many practical situations. In this study, we propose the fuzzy AHP method to overcome the weaknesses of conventional FMEA using the advantages of both fuzzy theory and AHP MCDM methods. The fuzzy theory is used to reduce the evaluation uncertainties on FMs, and the AHP method is used for determining relative weights of risk impacts based on inter‐relationships among FMs.

Our research group has previously published the results of FMEA and quality control measures to mitigate the failure modes for the commissioning process of the Halcyon (Varian Medical Systems, Palo Alto, CA) linear accelerator.^1^ This study highlighted unique FMs due to the new LINAC features and ring‐type gantry design and demonstrated the risk assessment approach recommended by the TG100 report. However, because of the heavy reliance on imaging with Halcyon, imaging involved many FMs in the commissioning process, and inter‐relationships among FMs were observed in high ranked FMs.

In this study, fuzzy AHP method is proposed to solve problems of traditional RPN‐based prioritization approach using both advantages of fuzzy theory and AHP MCDM method. First, we aimed to account the relative importance of risk factors. The AHP method can effectively reflect the difference of weights considering inter‐dependencies without complicated calculations. Second, fuzzy theory is used to reduce the uncertainties related to evaluators’ assessments due to lack of knowledge and inaccuracy. The fuzzy theory can provide meaningful representation of uncertainties in terms of intervals or fuzzy numbers. We implemented our fuzzy AHP‐based RPN method to the commissioning process of Halcyon linear accelerator.

In this manuscript, the fuzzy membership function and linguistic description used in this study was described in Section 2.1 for converting the numerical value into a fuzzy number. In Section 2.2, AHP method for calculating weights of risk factors and method to obtain two fuzzy RPNs were demonstrated. Then, the results from two fuzzy RPNs were compared and discussed.

## 2 METHODS

The Halcyon™ treatment delivery system introduced new design features that facilitate efficient and simplified workflow emphasizing the enhanced imaging capability. Compared to a conventional c‐arm gantry LINAC, it has several distinct features for image‐guided radiation therapy (IGRT), which are: enclosed design with a 100 cm diameter bore, use of an external laser for initial patient setup, omission of the light field and optical distance indicator, and mandatory IGRT‐based delivery.

**FIGURE 1:**
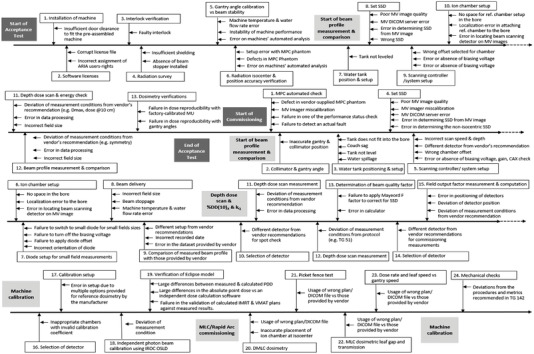
The summarized process map and failure modes for the acceptance testing and commissioning of Halcyon^1^

Our research group has previously published the results of FMEA for the acceptance testing and commissioning (ATC) for the Halcyon machine.^1^ A total of 38 sub‐processes (SPs) and 88 FMs were identified for the entire process map. (14 SPs and 34 FMs for the acceptance testing process and 24 SPs and 54 FMs for the commissioning process) The acceptance testing involved the installation of machine and software, verification with the machine performance check (MPC) phantom, automated software analysis, measurements of beam profile, beam energy, various dosimetric parameters, and MV imager checks. For commissioning process, the measurement of beam profiles, percent depth dose, beam quality, field output factors, reference dosimetry, verification of the beam model, and RapidArc commissioning with MLC and gantry rotation verification were involved. Figure 1 shows the summarized process map and failure modes for the acceptance testing and commissioning of Halcyon in our previous study.^1^

In this study, two fuzzy RPNs, called RPN_W_ and FRPN_W_, were calculated depending on the input values of O, S, and D from the evaluators. The RPN_W_, used numerical values, whereas the fuzzy values were used for FRPN_W_. The FRPN_W_ is helpful when evaluators have difficulty in rating the exact number of risk factors for evaluating FMs. Both RPNs were calculated by multiplying the weighted O, S, and D using the fuzzy AHP. The fuzzy membership function and linguistic description used in this study was described in Section 2.1, and the method to calculate the fuzzy RPNs was described in Section 2.2.

We confined our fuzzy AHP study to eight SPs from “1: MPC automated check” to “8: beam delivery” in the beam profile measurement and comparison section of the commissioning process. The FMs in SP1 to SP8 showed many inter‐relationships among FMs which cause significant variations of risk factors among evaluators and these FMs were identified top ranked FMs in our previous study. The fuzzy AHP method was used to adjust weights of risk factors considering dependencies among FMs that can cause a single or multiple events. The FMs in other SPs were considered independent, therefore equal weights of risk factors are applied to calculate RPN which is same as traditional RPN method. Similarly, many independent FMs were observed in the acceptance testing process. Moreover, SP7 to SP10 in an acceptance testing were duplicated in the commissioning process from SP3 to SP6. For this reason, we confined our study to eight SPs in commissioning process.

We had O, S, and D values from six medical physicists who were involved for the risk assessment in our previous study. However, four evaluating physicists who have participated in the initial evaluation in the previous study changed their scores of risk factors as they gained three years’ experience and knowledge on this machine. These modified scores were used for this study.

### 2.1 Fuzzy membership function and linguistic description

Fuzzy set theory, first introduced by Zadeh,^16^, ^17^ is a well‐known decision support tool that allows experts to handle the uncertainty in risk assessment. In fuzzy FMEA, the factors O, S, and D are evaluated in linguistic terms. This linguistic term is represented by a fuzzy membership function, which is defined by its central value and parameters such as the spread of the Gaussian, left and right end of the respective triangular, trapezoidal function, and so forth. These fuzzy membership functions represent a probability distribution. It is assumed that if the representation of the input risk factor is in probabilistic sense, the output risk distribution may reflect probabilistic information. In general, triangular or trapezoidal shape fuzzy membership functions have been widely used due to their simplicity.

**FIGURE 2:**
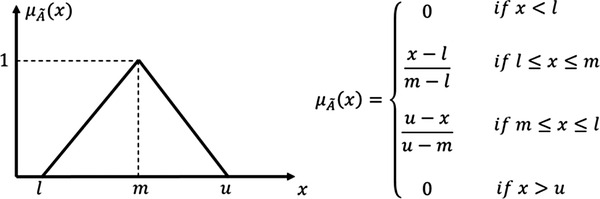
Fuzzy triangular number membership function

A fuzzy set $\tilde{A}$ can be defined mathematically by a membership function $\mu_{\tilde{A}}\left(x\right)$, which assigns each element *x* in the universe of discourse $X=\{x_{1},x_{2},\text{…},x_{n}\}$ a real number in the interval [0, 1]. Triangular and trapezoidal fuzzy numbers are the most commonly used fuzzy membership. In this study, we implemented a triangular fuzzy number for simplicity. The triangular fuzzy number as represented by three points $\tilde{A}=\;\left(l,m,u\right)$, which are lower value (*l*), middle value (*m*), and upper value (*u*), and membership function $\mu_{\tilde{A}}\left(x\right)$ describes the degree of x∈X belong to $\tilde{A}$ shown in Figure 2.

Our linguistic variables are categorized based on the descriptions of O, S, and D in the TG‐100 report. Table 1 shows the rank values based on the qualitative and frequency for O, qualitative and categorization for S, and estimated probability for D descripted in the TG‐100 report. The estimated probability of D is a probability of failure going undetected in percentage.

**TABLE 1:** Descriptions of the O, S, and D values in the TG‐100 report and fuzzy linguistic variable of three risk factors

	**Occurrence (O)**				**Severity (S)**				**Detectability (D) estimated probability of failure going undetected in percentage**	
**Rank**	**Qualitative**	**Frequency**	**Linguistic**	**Rank**	**Qualitative**	**Categorization**	**Linguistic**	**Rank**	**Estimated probability**	**Linguistic**
1	Failure unlikely	0.01%	VL	1	No effect		VL	1	0.01%	VL
2		0.02%		2 3	Inconvenience	Inconvenience	L	2	0.2%	L
3	Relatively few failures	0.05%	L					3	0.5%	
4		0.1%		4	Minor dosimetric error	Suboptimal plan or treatment	M	4	1.0%	M
5		<0.2%		5 6	Limited toxicity or tumor underdose	Wrong dose, dose distribution, location or volume	H	5	2.0%	
6	Occasional failures	<0.5%	M					6	5.0%	
7		<1%		7 8	Potentially serious toxicity or tumor underdose			7	10%	H
8	Repeated failures	<2%	H					8	15%	
9		<5%		9	Possible very serious toxicity or tumor underdose	Very wrong dose, dose distribution, location or volume	VH	9	20%	VH
10	Failures inevitable	>5%	VH	10	Catastrophic			10	>20%	

**FIGURE 3:**
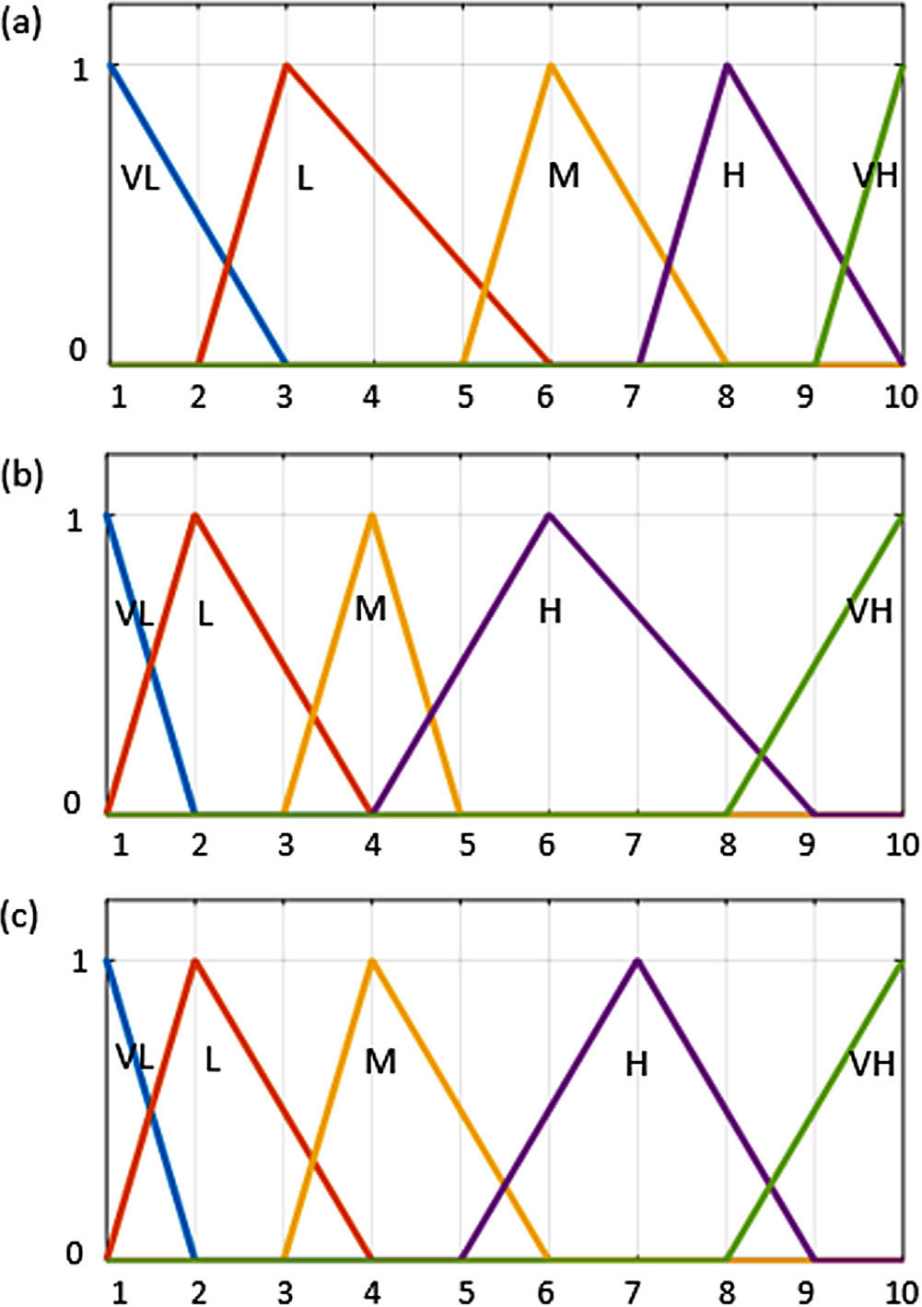
Fuzzy membership function for (a) occurrence, (b) detectability, and (c) severity

The set of linguistic criteria used in this study are Very Low (VL), Low (L), Moderate (M), High (H), and Very High (VH). These five levels of fuzzy linguistic scales are assigned to 1 to 10 points as a form of the membership functions shown in Figure 3. These five levels of fuzzy linguistic scales were determined based on the qualitative descriptions and estimated probability in TG‐100, and their corresponding fuzzy numbers were determined according to their rank values.

Since we have numerical scores of risk factors for evaluating FMs from previous study, these numbers were converted to the fuzzy numbers based on five levels of linguistic scales to simulate the situation where the linguistic scales were obtained from the evaluators. Once fuzzy numbers are obtained, the triangular fuzzy numbers for each FM were aggregated to combine the individual evaluator's opinion to form a group consensus opinion. The aggregation is performed by the following equation:

(1) $$\tilde{A}=\left(l,m,u\right)\;=\left(\min l_{n}^{k},\frac{1}{K}\sum_{k=1}^{K}m_{n}^{K},\max u_{n}^{k}\right)$$

where *k* represents the number of evaluators for *n* th FM. Fuzzy numbers need to be transformed into the crisp number. Such a transformation process is called defuzzification. The crisp number $\alpha_{\tilde{A}}$ corresponding to the aggregated fuzzy number is obtained by a centroid defuzzification method. For a triangular fuzzy number, the centroid defuzzification method can be simplified as shown in Equation (2).

(2) $$\alpha_{\tilde{A}}=\frac{l+m+u}{3}$$

This crisp number $\alpha_{\tilde{A}}$ is the defuzzied value of a risk factor. To calculate the fuzzy RPN, the process to obtain the $\alpha_{\tilde{A}}$ was performed separately for three risk factors, O, S, and D.

### 2.2 Fuzzy risk priority numbers

The fuzzy AHP method is used to calculate the RPN. Two fuzzy RPNs, RPN_W_ and FRPN_W_, are calculated by multiplying O, S, and D and weights of sub‐process (SP for O, S, and D for each FM. The RPN_W_ uses numerical values of risk factors from evaluators, whereas the FRPN_W_ uses fuzzy linguistic terms for risk factors. Since we already have the numerical O, S, and D values from our previous study, these numbers were used directly for the RPN_W_. The fuzzy linguistic terms for the FRPN_W_ were driven by converting numerical values to fuzzy terms based on the description of the triangular membership function in Section 2.1. This scenario is useful when evaluators evaluate FMs using linguistic terms.

The fuzzy AHP method is used to determine the weights of SPs for risk factors. In our AHP framework, the goal is to find relative weights of SPs for each risk factor, and evaluation criteria is the inter‐dependency among SPs. The evaluators assess the alternatives (SPs) with respect to criteria. Evaluators were asked to draw a causal diagram with arrows and fuzzy linguistic scales. The five levels of fuzzy linguistic scales were also used to evaluate the degree of relevance among SPs for AHP process. The set of linguistic scales are VL, L, M, H, and VH, and their corresponding triangular fuzzy numbers are described in Table 2. The symmetrical and uniform interval triangular functions were used to obtain the weights of O, S, and D, because the goal of AHP method is to find the relative importance among SPs. These fuzzy linguistic scales are same for all risk factors.

**TABLE 2:** The AHP linguistic scales and corresponding triangular fuzzy numbers

**Linguistic scale**	**Triangular fuzzy numbers**	**Inverse of triangular fuzzy number**
Very low importance (VL)	(1, 1, 3)	(1/3, 1, 1)
Low importance (L)	(1, 3, 5)	(1/5, 1/3, 1)
Medium importance (M)	(3, 5, 7)	(1/7, 1/5, 1/3)
High importance (H)	(5, 7, 9)	(1/9, 1/7, 1/5)
Very high importance (VH)	(7, 9, 10)	(1/10, 1/9, 1/7)

We instructed evaluators when they evaluate the inter‐relationship among SPs for each risk factor using fuzzy linguistic scales. O is based on the causal influence of one SP on the occurrence of another SP, S indicates the influence of the degree of severity of one SP on that of another SP, and D indicates the feedback where the detectability of later SPs results in an increase in the detectability of earlier SPs. We defined the degree of S as an unnecessary time delay in completing the commissioning process for the machine setup. The arrows were therefore directed toward later SPs for O and S, and toward earlier SPs for D. The starting point (the non‐arrow side) of the arrows has a priority over the ending points of the in the causal diagram. The rules above the evaluators’ responses with linguistic terms were converted to triangular fuzzy numbers, and subsequently our AHP pairwise comparison matrices were constructed.

The pairwise comparison matrix ($\tilde{A_{k}}$) is shown in Equation (3), where ${\tilde{a}}_{ij}^{k}$ indicates the importance of *i* criterion over *j*, of the *k* evaluator, expressed by fuzzy triangular numbers. For example, ${\tilde{a}}_{12}^{1}$ represents the first evaluator's evaluation of the importance of SP1 over SP2. In the case where SP1 is moderately more important than SP2, fuzzy number will be (3, 5, 7), whereas ${\tilde{a}}_{21}^{1}$would be (1/7, 1/5, 1/3). When the importance is revered, fuzzy numbers were transposed in the matrix. For example, ${\tilde{a}}_{ij}^{k}$ of the lower left side of the matrix have an inverse of ${\tilde{a}}_{ij}^{k}$ due to the high priority of O and S in earlier SPs, whereas ${\tilde{a}}_{ij}^{k}$ of the upper right side of the matrix have an inverse of ${\tilde{a}}_{ij}^{k}$ due to the high priority of D in later SPs. The fuzzy number (1, 1, 1,) would be entered when two SPs are equally important, and *n* is the number of SPs in Equation (3).

(3) $$\tilde{A_{k}}=\left[\begin{array}{c} 1\\ {\tilde{a}}_{21}^{k}\\ \vdots \\ {\tilde{a}}_{n1}^{k} \end{array}\begin{array}{c} {\tilde{a}}_{12}^{k}\\ 1\\ \vdots \\ {\tilde{a}}_{n2}^{k} \end{array}\begin{array}{c} \cdots \\ \cdots \\ \cdots \\ \cdots \end{array}\begin{array}{c} {\tilde{a}}_{1n}^{k}\\ {\tilde{a}}_{2n}^{k}\\ \vdots \\ 1 \end{array}\right]$$

These fuzzy numbers in pairwise matrices from different evaluators were aggregated using Equation (1). Once the aggregate fuzzy pairwise comparison matrices were calculated, a priority vector of the above pairwise matrix need to be calculated. In this study, the fuzzy extent analysis method by Chang^23^ was used to calculate the crisp weights from our pairwise matrix. This method is one of the most popular fuzzy AHP method, which is summarized as follows. First, the fuzzy synthetic extent is for each SP calculated. The value of fuzzy synthetic extent with respect to *𝑖*𝑡ℎ SP can be determined by using the algebraic operations on triangular fuzzy numbers as follows:

(4) $$S_{i}=\sum_{j=1}^{n}{\tilde{M}}_{ji}⊗{\left[\sum_{i=1}^{n}\sum_{j=1}^{n}{\tilde{M}}_{ji}\right]}^{-1}$$

where ${\tilde{M}}_{ji}$ is the triangular fuzzy number. To obtain $\sum_{j=1}^{n}{\tilde{M}}_{ji}$ and $\sum_{i=1}^{n}\sum_{j=1}^{n}{\tilde{M}}_{ji}$, the fuzzy addition operation is performed for the entire triangular fuzzy number as shown in Equations (5) and (6).

(5) $$\sum_{j=1}^{n}{\tilde{M}}_{ji}=\left(\sum_{j=1}^{n}l_{i},\sum_{j=1}^{n}m_{i},\sum_{j=1}^{n}u_{i}\right)$$

(6) $$\sum_{i=1}^{n}\sum_{j=1}^{n}{\tilde{M}}_{ji}=\left(\sum_{i=1}^{n}l_{i},\sum_{i=1}^{n}m_{i},\sum_{i=1}^{n}u_{i}\right)$$

**FIGURE 4:**
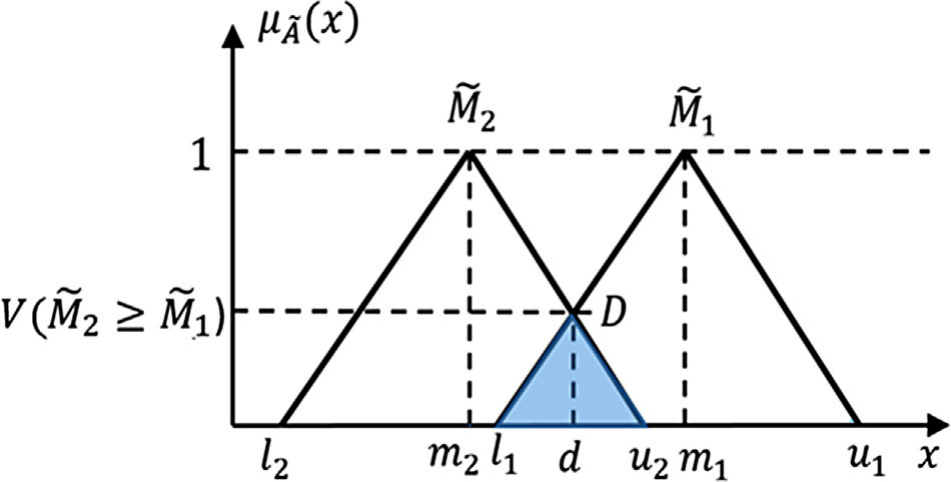
The intersection point between ${\tilde{M}}_{2}$ and ${\tilde{M}}_{1}$

Next, the degree of possibilities of each SP is calculated by comparing of fuzzy synthetic extent, ${\tilde{M}}_{i}$. The degree of possibility (*V*) of two membership functions, which are ${\tilde{M}}_{2}=\;(l_{2},m_{2},u_{2})\;\geq \;\;{\tilde{M}}_{1}=\;(l_{1},m_{1},u_{1})$, is defined as:

(7) $$\begin{array}{ccc} V({\tilde{M}}_{2}\geq {\tilde{M}}_{1}) & = & hgt\left({\tilde{M}}_{2}\cap {\tilde{M}}_{1}\right)={\tilde{M}}_{2}\left(d\right)\\ & & =\left\{\begin{array}{c} 1\\ 0\\ \frac{l_{1}-u_{2}}{\left(m_{2}-u_{2}\right)-\left(m_{1}-l_{1}\right)} \end{array}\begin{array}{c} \;if\;m_{2}\geq m_{1}\\ \;if\;l_{1}\geq u_{2},\\ \;otherwise \end{array}\right. \end{array}$$

where *d* is the ordinate of the highest intersection point D located between ${\tilde{M}}_{2}$ and ${\tilde{M}}_{1}$ in Figure 4. To compare ${\tilde{M}}_{1}$ and ${\tilde{M}}_{2}$, both $V({\tilde{M}}_{2}\geq$ ${\tilde{M}}_{1}$) and $V({\tilde{M}}_{1}\geq$ ${\tilde{M}}_{1}$) are calculated. Then, the probability degree where each fuzzy number is greater than 𝑘 fuzzy membership number $M_{i}\left(i=1,2,\text{…},k\right)$ is defined as

(8) $$\begin{array}{ccc} V(\tilde{M}\geq {\tilde{M}}_{1},{\tilde{M}}_{2},\text{…},{\tilde{M}}_{k}) & = & min\;V(\tilde{M}\geq {\tilde{M}}_{i}),\\ i & = & 1,2,\text{…},k \end{array}$$

Finally, the weights of each SP are determined by Equation (9).

(9) $$W\;={\left(w_{1},w_{2},\text{…},w_{n}\right)}^{T}$$

where $w_{i}$ is the normalized weight values obtained by

(10) $$w_{i}=\frac{V\left({\tilde{M}}_{i}\geq {\tilde{M}}_{j}\right)}{\sum_{k=1}^{n}V\left({\tilde{M}}_{i}\geq {\tilde{M}}_{j}\right)}$$

The $w_{i}$ is weight values of SPs of a risk factor, and the process to obtain the $w_{i}$ is performed separately for each O, S, and D. Finally, the fuzzy RPN for each FM in a certain SP was calculated by multiplying weights of SP of risk factors and risk factor values as follows:

(11) $${\mathrm{RPN}}_{w}=\;Ow_{iO}\times Sw_{iS}\times Dw_{iD}$$

To obtain the crisp numbers of O, S, and D from the membership function for FRPN_W_, the aggregation and defuzzification of fuzzy numbers were performed, described in the Equations (1) and (2) in Section 2.1. The numerical O, S, and D values from our previous study were used to calculate RPN_W_.

## 3 RESULTS

**FIGURE 5:**
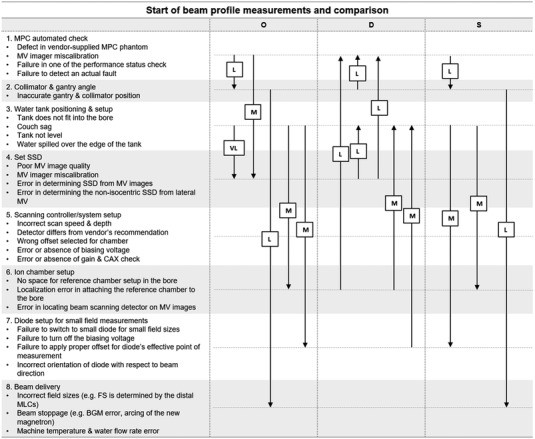
The sample causal diagram for O, S, and D to represent interrelations among sub‐processes

**FIGURE 6:**
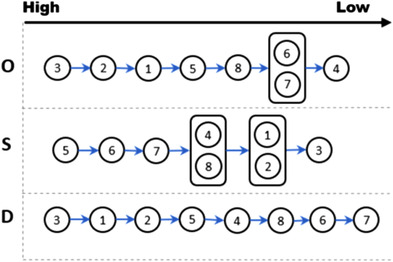
The hierarchy of priority for O, S, and D of fuzzy analytic hierarchy process approach

A sample causal diagram for O, S, and D to represent interrelations among a total of eight SPs in a beam profile measurement of the Halcyon commissioning process is presented in Figure 5. It can be seen that the arrows of O and S proceeded in time sequence, while D proceeded in inverse of time sequence because O and S are trigger effects and D represents a feedback loop. Figure 6 represents the hierarchy of priority for O, S, and D based on weighting values as a result of fuzzy AHP. The SP3, SP2, and SP1 have a higher importance than other SPs because these SPs cause an occurrence of other SPs. Likewise, SP3, SP1, and SP2 have a higher importance for S due to an increased severity when previous FMs occur. In this commissioning process, an increased S is an unnecessary time delay in completing the commissioning process. It is not related to dose modeling and delivery to future patients because the Halcyon beam model is pre‐configured and cannot be changed. For D, earlier SPs have lower importance than later SPs because FMs were more likely to be detected at later SPs. The SPs that have the highest importance is an independent SP because there is no possibility to detect at later SPs. The least important SP have a lot of inter‐dependencies due to the increased possibility to detect in other SPs. Since the time sequence dependency is a major factor for risk factors, earlier SPs have lower importance than later SPs. The SPs shown inside a box indicate SPs which have the same importance.

The top five FMs ranked by traditional RPN and fuzzy RPN_W_ are shown in Table 3. For traditional RPN‐based analysis, the top two highest ranked FMs among the top five FMs arise from SP4, that is, errors in determining the non‐isocentric SSD (e.g., SSD of 90 cm) and SSD from MV images (e.g., inter‐ and intra‐observer error). The next top ranked FM arises from SP6, that is, error in locating field chamber on MV images. The fourth and fifth ranked FMs arise from SP1, that is, failure to detect an actual fault in initial phantom setup and SP4, that is, poor MV image resolution. For fuzzy RPN_W_‐based analysis, the top two FMs are the same as those obtained from the traditional RPN. The weights of risk factors did not change the rank of the two top FMs, because the RPNs of the top two FMs were much higher than those of the other FMs in traditional FMEA. The next top ranked FMs arise from SP3, that is, couch sag and SP1. These SPs have higher weights of O and D than the others described in Figure 6.

**TABLE 3:** Top five traditional RPN and fuzzy RPN_W_ ranking

**Traditional RPN**				**Fuzzy** $\mathrm{RP}N_{W}$		
**Rank**	**SP**	**FMs**	**RPN**	**SP**	**FMs**	$\mathrm{RP}N_{W}$
1	4	Error in determining the non‐isocentric SSD	95.04	4	Error in determining the non‐isocentric SSD	0.1398
2	4	Error in determining SSD from MV images	94.24	4	Error in determining SSD from MV images	0.1386
3	6	Error in locating field chamber on MV images	55.30	3	Couch sag	0.1037
4	1	Failure to detect an actual fault	54.00	1	Failure to detect an actual fault	0.0982
5	4	Poor MV image resolution	46.24	3	Water slopped over the edge of the tank	0.0842

**FIGURE 7:**
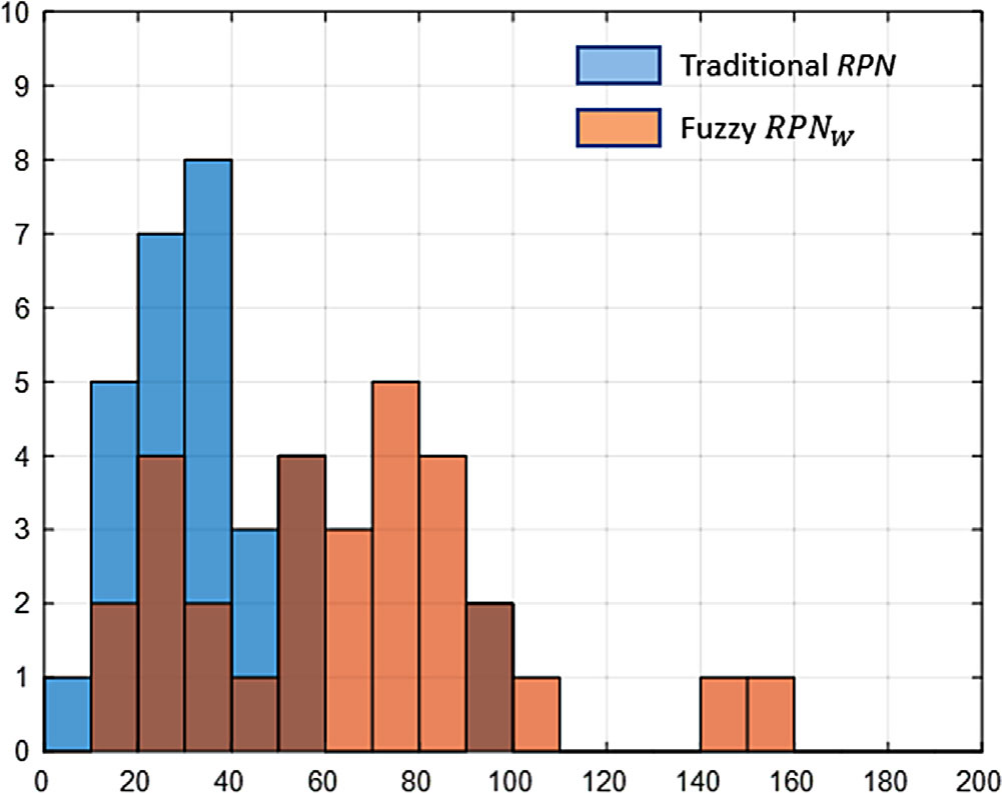
The risk priority number (RPN) distribution of traditional RPN and fuzzy RPN_W_ approaches

Figure 7 shows the RPN distributions of traditional RPN and fuzzy RPN_W_  in the same scale. The range of fuzzy RPN_W_ is wider compared to that for traditional RPN. Except top two FMs, traditional RPNs are ranged from 0 to 60, while fuzzy RPN_W_ are distributed from 10 to 110. The shaded colors are the overlapping of two bars.

The top five FMs ranked by fuzzy FRPN_W_ are shown in Table 4. Since the FRPN_W_ uses fuzzy terms as opposed to RPN_W_ which uses numerical values from evaluators, the fuzzy uncertainty could be evaluated by comparing the rank from RPN_W_ in Table 3. For FRPN_W_, the rank of the top two FMs and the third and fourth FMs are switched compared to those obtained from the fuzzy RPN_W_. The fifth ranked FM also arises from SP3, however the ranked FM is changed. The differences between the two RPN rankings are due to inherent fuzzy uncertainty. Since fuzzy linguistic variables consist of five levels from VL to VH instead of a 10‐point scale, if the value of a risk factor falls at the wider spread of the fuzzy membership function, the uncertainty will be larger, and smaller when the value falls at the narrow spread of the membership function. It is known that when the number of evaluators is small, and the evaluation difference of each expert is large, random uncertainty has a greater effect on the evaluation results than fuzzy uncertainty. In our previous study, the maximum deviation in traditional O, S, and D values between evaluators was six out of a 10‐point scale, and this maximum deviation was seen for O for the second ranked FM, S and D for the third ranked FM, and S for the fourth and fifth ranked FMs. Therefore, it is reasonable to assume that the differences between the two RPN rankings are mainly due to deviations in the traditional O, S, and D values submitted by the evaluators.

**TABLE 4:** Top five fuzzy FRPN_W_ ranking

**Rank**	**SP**	**FMs**	**O**	**S**	**D**	***w***_**O**_	***w***_**S**_	***w***_**D**_	$\mathrm{FRP}N_{W}$
1	4	Error in determining SSD from MV images	6.190	4.919	3.514	0.100	0.136	0.109	0.1587
2	4	Error in determining the non‐isocentric SSD	6.190	4.919	3.119	0.100	0.136	0.109	0.1407
3	1	Failure to detect an actual fault	1.325	7.703	3.606	0.209	0.074	0.193	0.1098
4	3	Couch sag	6.127	2.911	2.980	0.134	0.09	0.151	0.0970
5	3	Tank not levelled	7.265	1.569	3.145	0.113	0.167	0.131	0.0887

Considering all results of traditional and fuzzy FMEA, the two most important FMs included errors in determining the non‐isocentric SSD (e.g., SSD of 90 cm) and SSD from lateral MV images (e.g., inter‐ and intra‐observer error) because of the unique feature of the ring gantry LINAC. These two RPNs in traditional FMEA were adjusted based on fuzzy AHP, making other FMs that cause these failures obtain larger RPNs in earlier SPs of the commissioning process, for example, MV imager miscalibration in SP 1. Similarly, the RPNs of FMs in SP3 were increased in fuzzy FMEA because these may trigger or increase the S at later FMs in SP 6, for example, error in locating field chamber in MV images.

## 4 DISCUSSION

In this study, we proposed the fuzzy AHP‐based RPN method in FMEA and results showed a lot of benefits of this method. First, evaluation uncertainties due to imprecise knowledge from evaluators can be modeled as a form of membership function. An assignment of values for O, S, and D was substantially subjective and influenced by evaluators’ experience, especially when dealing with new type of machines and procedures. Our previous study based on traditional RPN approach also showed large deviations (≤6 out of 10) of O, S, and D values among evaluators in high ranked FMs. Fuzzy theory uses the limited evaluation scales as a form of a membership function, values of O, S, and D are more consistent among evaluators. Then, aggregation and defuzzification methods are used to obtain the final crisp number. Second, a wide range of fuzzy RPN can be achieved. One of the most important steps in FMEA procedure is to prioritize FMs to assign the limited resources to the most serious FMs. Duplicate or similar RPN values are difficult to judge considering subjectivity and inaccuracy of O, S, and D values in assessment in traditional RPN. Based on the priority of each FM, we developed corrective actions only for top five ranked FMs to maximize efficiency. Third, our fuzzy AHP‐based RPN provides the benefits of assigning different weights of risk factors depending on the dependency among FMs and accepting fuzzy numbers for evaluators in cases where there are no prior statistical data. It also provides a flexibility in evaluating FMs using different weighting approaches. The considerable discrepancies were found among opinions of the experts in the process of FMEA. Some team members considered the worst case that could happen if the FM occurs, while others considered the most probable consequences on the patient, taking into consideration their experience.^24^ Using our fuzzy AHP approach, we can also simulate worst‐case scenarios by prioritizing FMs by focusing on consequences. In this study, we prioritize FMs focusing on the root cause of FMs, addressing the cause of the problem by putting more weight on earlier SPs of the commissioning process. In a consequence‐based approach, it can be simply done by putting weights on the ending points (the arrow side) of the arrows over the starting points (the non‐arrow side) of the arrows in the causal diagram.

Despite many advantages of our fuzzy AHP‐based RPN approach, the fuzzy RPN has several limitations. First, fuzzy theory has inherent uncertainties because it deals with a range of values, and subsequently the accuracy could be compromised depending on fuzzy aggregation methods, design of membership function, and large variation among evaluators. Therefore, fuzzy FMEA must be executed by qualified team members, and numerical values of O, S, and D are also allowed when evaluators are confident to provide a crisp number for each risk factor of each FM. In our study, qualified team members are the professionals who were involved our initial study and continued their practice in Halcyon machine. Second, the necessity to draw the causal diagram can be additional work for evaluators and a limitation of the proposed approach. However, most evaluators thought the causal diagram helpful to take a comprehensive approach by clarifying all possible dependencies among SPs. It also helps evaluators take a unified evaluation when they assigned O, S, and D values by focusing on the FM itself without considering other factors.

The most important part of performing FMEA is to generate a well‐organized comprehensive process map and FMs by team members, and to make sure that FMs are not inter‐dependent, which may cause a different interpretation among evaluators in determining O, S, and D values. For example, “setup differ from vendor recommendation” and “incorrect data recorded” in SP9: comparison of measured beam profile with those provided by Varian may be misleading because evaluators may correlate these failures to water tank positioning/scanning controller/ion chamber setup in SP3, SP5, and SP6 in the commissioning process. These FMs may cause large discrepancies among evaluators due to misunderstanding or misconception. For this reason, the SP9 was not included in our fuzzy AHP study because it may cause large discrepancies among evaluators. We revised FMs in the commissioning process of our previous study to avoid possible confusion for interpreting FMs. The revised FMEA was implemented by our other network hospitals when they are performing ATC process of same machine. In addition, we recently installed another ring type of gantry LINAC, RefleXion (RefleXion Medical, Hayward CA) It shares a lot of similar features as Halcyon LINAC in ATC process, including the use of image in locating beam scanning detector, non‐intuitive gantry and collimator position, and deviation of measurement conditions from the protocol (e.g., non‐standard SSD setting), therefore it shares many FMs. Based on the knowledge that our team member obtained from previous study, we particularly paid attention to top ranked FMs when we are performing ATC process.

We allowed changes of the scores of risk factors from the evaluators who were involved the initial evaluation. Two FMs that showed significant changes in scores were “machine temperature and water flow rate error” in SP5 for the acceptance test and “incorrect field size and gantry angle” in SP8 for the acceptance test. From our previous study, “machine temperature and water flow rate error” was the third highest‐ranked FM for acceptance because the chilled water supply for the Halcyon machine was shared with that for an existing adjacent LINAC. Sharing the supply of chilled water led to overheating of the adjacent LINAC that is in clinical operation. During this period, rebalancing of the chilled water supply between the Halcyon LINAC and the adjacent LINAC was required to solve the issue. However, this issue persisted, and it caused overheating of Halcyon LINAC leading to a machine down situation for several days. This FM earned more S scores. Increased O values were found in “incorrect field size and gantry angle.” Several MV images were required to set up the ion chamber at gantry angle 90. After physicists confirmed the chamber setup, it is often forgotten to set a gantry angle zero for a measurement due to the ring gantry design of the Halcyon LINAC. Evaluators assigned increased O values to this FM based on their experiences during monthly and annual QAs.

We have had several years of clinical experience with the Halcyon machine since it was first installed in our hospital. Since then, various FMEA projects were performed on this machine including for the IGRT process and for Ethos, an adaptive radiation therapy system for Halcyon. For the IGRT process, a lot of inter‐relationships were observed due to a heavy reliance on the imaging system and treatment couch limitations. For example, a proper initial patient setup may lead to both incorrect interpretation of patient position and repeated re‐positioning after imaging due to the lack of rotational couch motion. It may also cause a potential collision of the patient and/or immobilization devices with the treatment bore. In the case of the Ethos project, it was a prospective study where we had no historical data and direct experience with this system. Evaluators might presume the Ethos adaptive planning procedure is similar to earlier adaptive planning approaches and estimate the risks based on their previous experience. The fuzzy FMEA approach can be a suitable option for these FMEA cases.

Recently, this fuzzy theory became a powerful tool when it was combined with MCDM methods. MCDM is the techniques that have recently been gaining extraordinary popularity and wide applications and there are the methods for making decisions when multiple criteria need to be considered together, in order to rank or choose between the alternatives being evaluated. The popular MCDM methods are technique for order performance by similarity to ideal solution (TOPSIS),^25^, ^26^, ^27^ AHP^23,28^ and analytic network process (ANP).^29^, ^30^ TOPSIS is a compensatory aggregation method that allows trade‐offs between criteria, which minimizes the cost criteria and maximizes the benefit criteria for a decision‐making. AHP is one of the well‐known multi‐criteria decision‐making techniques and provides a strong and flexible technical approach for robust decision‐making, which supports a prioritized decision‐making. While the AHP decomposes a decision problem in the form of a hierarchy of independent elements, the ANP replaces hierarchies with networks and makes it possible to perform structural decisions that involve functional dependencies. In this study, we used a hierarchical structure of AHP to reflect directional impacts such as causal influence and feedback loop rather than using the complicated and time consuming ANP method.

## 5 CONCLUSION

FMEA has been used for examining potential failures in a process and can be used as an important safety analysis tool. However, a risk assessment in FMEA by traditional RPN analysis often suffers some limits such as same relative importance between risk factors, production of many repeated RPN at the bottom of the scale, and subjective assessments of the FMs by experts. We have proposed a fuzzy AHP‐based RPN to overcome these weaknesses of traditional RPN‐based failure prioritization. This study has demonstrated the feasibility of the use of a fuzzy AHP‐based RPN to perform comprehensive analysis and prioritization of FMs. We believe that this is the first study on the implementation of fuzzy AHP‐based RPN for the risk assessment in radiation treatment procedures. The risk analysis using fuzzy AHP can be improved and/or refined based on the department's specific workflow and clinical preferences taking various priority weighting approaches into account.

## CONFLICT OF INTEREST

The authors declare no conflict of interest.

## AUTHOR CONTRIBUTIONS

Conception and design of study by Jina Chang and interpretation of data by Siyoung Jang and Ron Lalonde, and refinement of the manuscript by Saiful M. Huq. All coauthors reviewed and efited the manuscript.
